# A clinical decision model based on machine learning for ptosis

**DOI:** 10.1186/s12886-021-01923-5

**Published:** 2021-04-09

**Authors:** Xuefei Song, Weilin Tong, Chaoyu Lei, Jingxuan Huang, Xianqun Fan, Guangtao Zhai, Huifang Zhou

**Affiliations:** 1 Department of Ophthalmology, Ninth People’s Hospital, Shanghai Jiao Tong University School of Medicine, Shanghai, China; 2 Shanghai Key Laboratory of Orbital Diseases and Ocular Oncology, Shanghai, China; 3 School of Electronic Information and Electrical Engineering, Shanghai Jiao Tong University, Shanghai, China; 4 Shanghai Jiao Tong University School of Medicine, Shanghai, China

**Keywords:** Ptosis, Clinical decision making, Computer-assisted surgery, Machine learning

## Abstract

**Background:**

To establish a decision model based on two- (2D) and three-dimensional (3D) eye data of patients with ptosis for developing personalized surgery plans.

**Methods:**

Data of this retrospective, case-control study was collected from March 2019 to June 2019 at the Department of Ophthalmology, Shanghai Ninth People’s Hospital, and then the patients were followed up for 3 months. One hundred fifty-two complete feature eyes from 100 voluntary patients with ptosis and satisfactory surgical results were selected, with 48 eyes excluded due to any severe condition or improper collection and shooting angle. Three experimental schemes were set as follows: use 2D distance alone, use 3D distance alone, and use two distances at the same time. The five most common evaluation indicators used in the binary classification problem to test the decision model were accuracy (ACC), precision, recall, F1-score, and area under the curve (AUC).

**Results:**

For diagnostic discrimination, recall of “3D”, “2D” and “Both” schemes were 0.875, 0.875 and 0.938 respectively. And precision of the three schemes were 0.8333, 0.7778 and 1.0000 for the surgical procedure classification. Values of “Both” scheme that combined 2D and 3D data were the highest in two classifications.

**Conclusions:**

In this study, 3D eye data are introduced into clinical practice to construct a decision model for ptosis surgery. Our decision model presents exceptional prediction effect, especially when 2D and 3D data employed jointly.

## Background

Ptosis, the lowering of the eyelid below the normal position, occurs because of multiple causes, including myogenic, neurogenic, aponeurotic, mechanical, or traumatic [1, 2]. Severe ptosis might exhibit an impact on visual development, especially in infants and adolescents. Besides, it might become a psychosomatic disease due to its subsequent social problems caused by abnormal behavior and posture. Nowadays, surgery is the primary treatment for ptosis [3]—levator muscle resection and frontalis suspension are the two most common surgical methods. The former is mainly applicable to patients with adequate strength of the levator muscle, while the latter applies to a large subset of patients with severe ptosis [4]. After several years of development, both techniques of operations have been sufficiently mature to facilitate conjoint fascial sheath and other operations [5, 6]. Furthermore, attempts to achieve an eyelid lifting effect with eyelid implants are reported previously [7].

However, the choice of surgery method for ptosis depends on specific cases, which is prone to divergence among practitioners [8], resulting in deviations of patients’ expectations. It might also result in an embarrassing situation where the doctors are satisfied with the surgery results while the patients are not [9]. Typically, the two operation methods differ in the strength of the levator muscle, which is usually 3 or 4 mm. If the value is larger than the threshold, the levator muscle resection is performed; otherwise, the frontalis suspension is operated [10]. However, as the value is mainly determined by the analysis of the eye parameters, there are some inevitable operational problems, including low patient coordination and manual measurement. Therefore, the surgeons tend to believe in their own experiences rather than relying on the measurement data. Hence, reliable rules to guide the application are yet lacking, allowing the choice of surgery based on the doctor’s ability [11]. Such a doctor’s growth path is common in the clinical application; however, maximizing the effectiveness of the treatment is based on its theoretical suitability to the patients and not vice versa—especially when the treatment differs in principle qualitatively than quantitively. Therefore, the pre-determination of the treatment plan is essential in both theory and practice.

Besides, different from most medical operations for anatomical restoration, ptosis surgery also involves aesthetic considerations [12]. As a physical and mental disease that influences patients’ social interactions, the evaluation of patients and their families on the effect of surgery is more important than that of the doctors. Therefore, we should assess the surgical effect according to the evaluation of patients in the real clinical environment instead of the measurement of numbers. Taken together, while studying the decision-making problem of the surgical plan, it is optimal to set the actual value of the outcome variable in the decision-making model based on the patients’ evaluations.

With the development of structured light scanning [13, 14] and face recognition technology [15], eye information can be obtained rapidly and accurately. Both linear distance and surface distance not only improve the data dimension and accuracy but also realize the repeatability and traceability of the data measurement, according to requirements of clinical trials. Herein, we proposed to use structured light scanning technology to extract facial feature points and measure the plane distance and surface distance between multiple key points of the eye. This would restore the conventional simplified one-dimensional decision-making of ptosis surgery to the current 3D data processing problem, aiming to provide comprehensive eye data of other dimensions for surgery-related decisions. Therefore, we established a decision-making model by collecting the two-dimensional (2D) and 3D eye data of patients with ptosis, which would support the doctors in designing a personalized approach for ptosis surgery.

## Methods

### Patients and study design

The experimental data were obtained from 100 patients with ptosis at the Department of Ophthalmology, Shanghai Ninth People’s Hospital, who voluntarily faced structured light cameras for the collection of facial information and allowed retrieval of surgery. The recruited 100 patients had satisfactory surgery results after following up for 3 months.

**Fig. 1:**
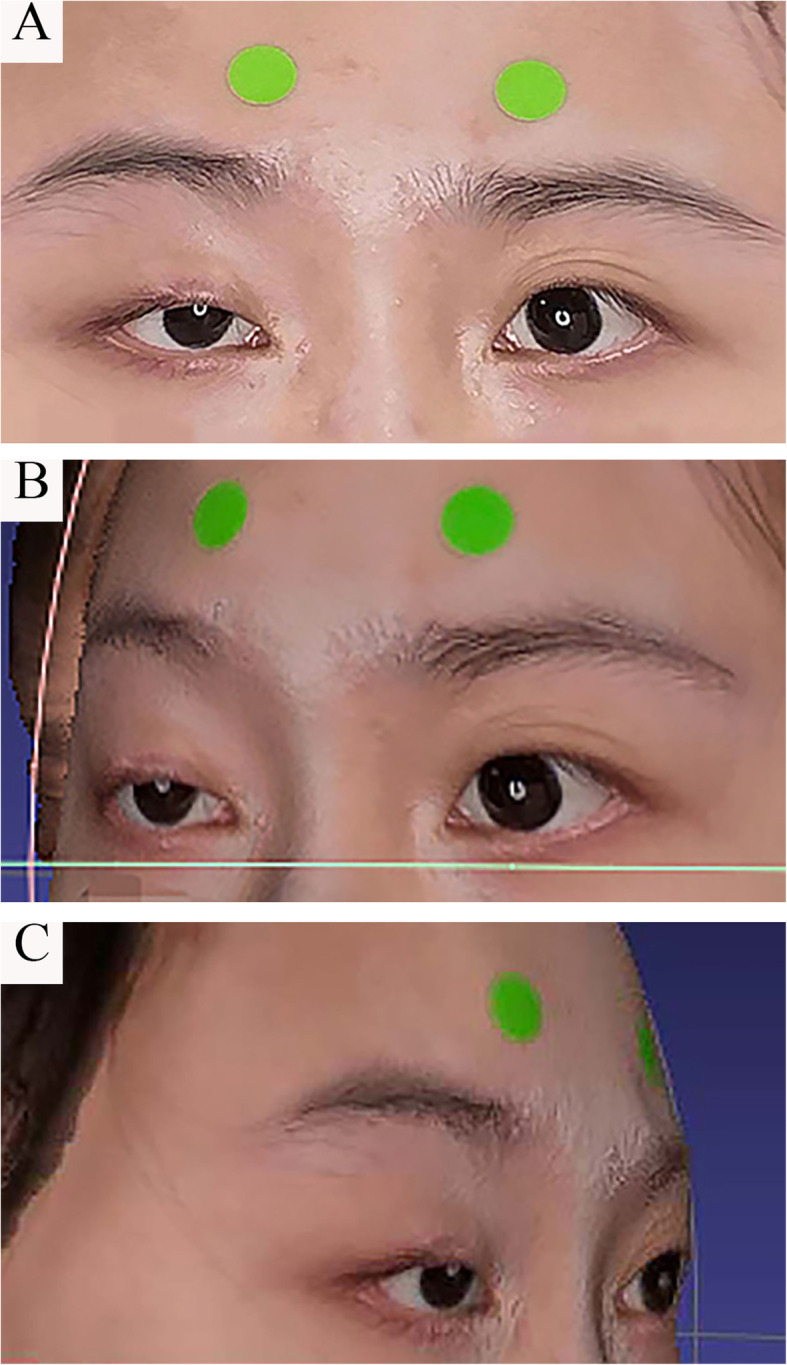
2D image and 3D face model of patients with ptosis. **a**. The 2D image of a patient scanned by the structured light camera; **b**, **c**. An OBJ 3D model obtained by 3D reconstruction of about 120,000 key points of the face collected by bellus3d according to the structured light camera

The data of each patient was composed of a single 2D photo and an OBJ file format 3D model obtained by 3D reconstruction of about 120,000 key points of the face collected by bellus3d according to the structured light camera (Fig. 1).

**Fig. 2:**
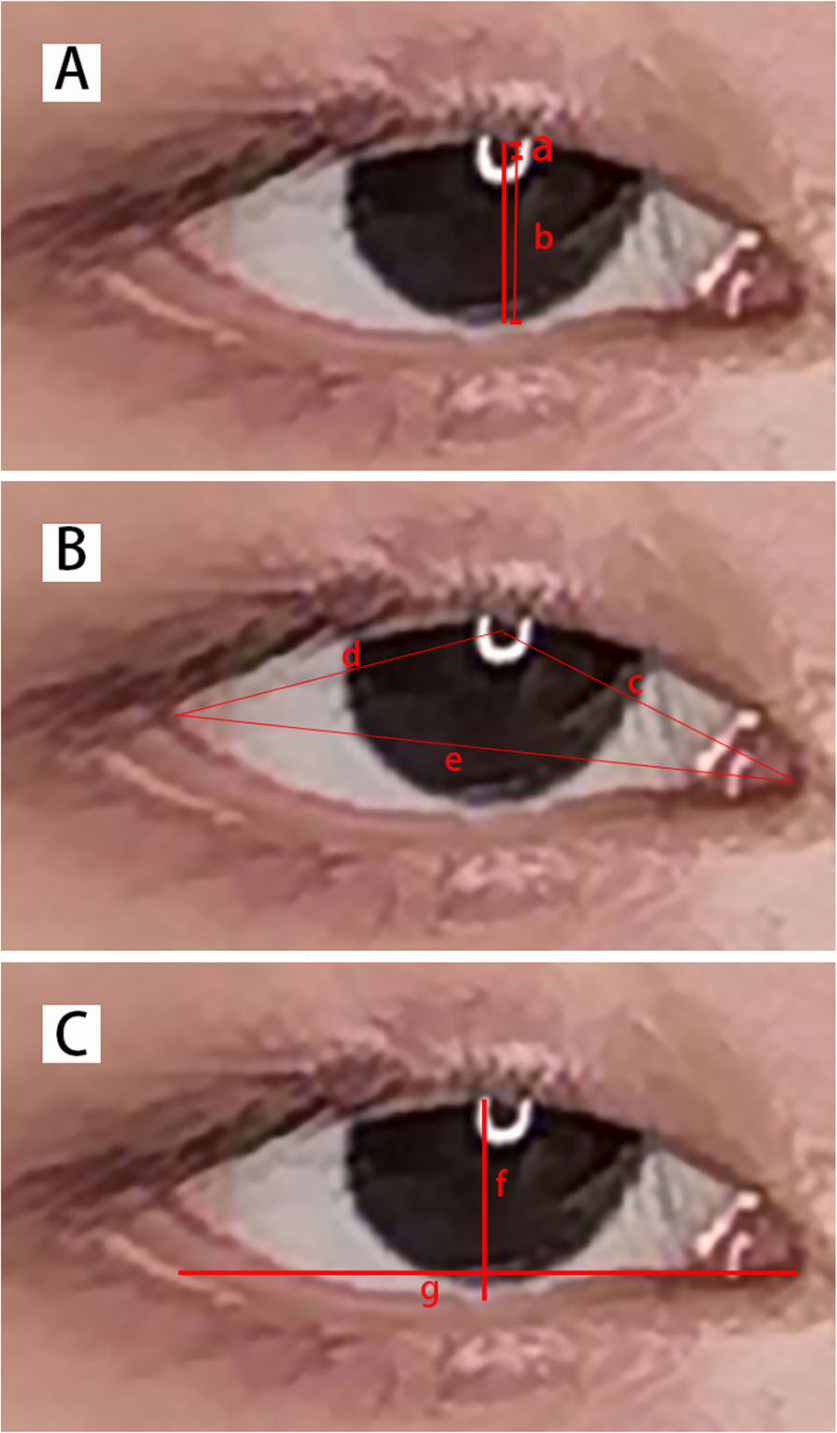
Seven key eye distances manually marked for ptosis patients. **a**. Margin reflex distance 1 (MRD1): The distance from the upper eyelid margin to the corneal light reflex. **b**. MRD2: The corneal light reflex to the lower eyelid margin. **c**. The distance between the inner canthus and the center of the eye. **d**. The distance between the epicanthus and the center of the eye. **e**. The distance between the inner and the outer canthus. **f**. The length of palpebral fissure: The horizontal distance between inner and outer canthus. **g**. The width of palpebral fissure: The maximum distance between the upper and the lower eyelids

The clinical data of the patients with ptosis were processed as follows:

1. Data cleaning: We conducted a rigorous screening of the 200 eyes to exclude any severe condition or improper collection and shooting angle, so that the key points of the eye could be obtained in the 2D pictures and 3D reconstruction models. Finally, 152 complete features were obtained from the patient’s eyes at a distance.
2. Data classification: Based on the tracking records of the patients’ operations, the 152 eyes were categorized into 44 eyes without surgery, 61 eyes with levator muscle resection, and 47 eyes with frontalis suspension. These categorizations prompted three types of clinical, surgical decision-making results.
3. Manual annotation: The manually annotated key data of the classified patients’ eyes were divided into 2D and 3D. We selected and manually marked seven key eye distances that were most critical for orbital diseases (Fig. 2). And the standard database format required by the subsequent decision model was obtained after comprehensive sorting to facilitate the input of the model.

To obtain the actual size of the picture, we placed two discs with a diameter of 퐷푟푒푎푙 10.0푚푚 mm on the forehead of the patient. The label was applied and laid flat and parallel to the camera lens to minimize the errors caused by the tilt. In order to facilitate the distinction, this disc was marked in green. Assuming that the circle in the patient’s eye image is Di퐷푖푚푎푔푒 pixels, the zoom ratio of the photo size corresponding to the actual size is: S = Di/10 mm; when the pixel value was measured as 7 distances, we calculated the true distance of these keys corresponding to the S ratio. In addition, in order to calculate the distance more accurately, we recorded multiple diameter distances of the two wafers in different directions at the same time during the operation, averaged them, and obtained the ratio.

**Fig. 3:**
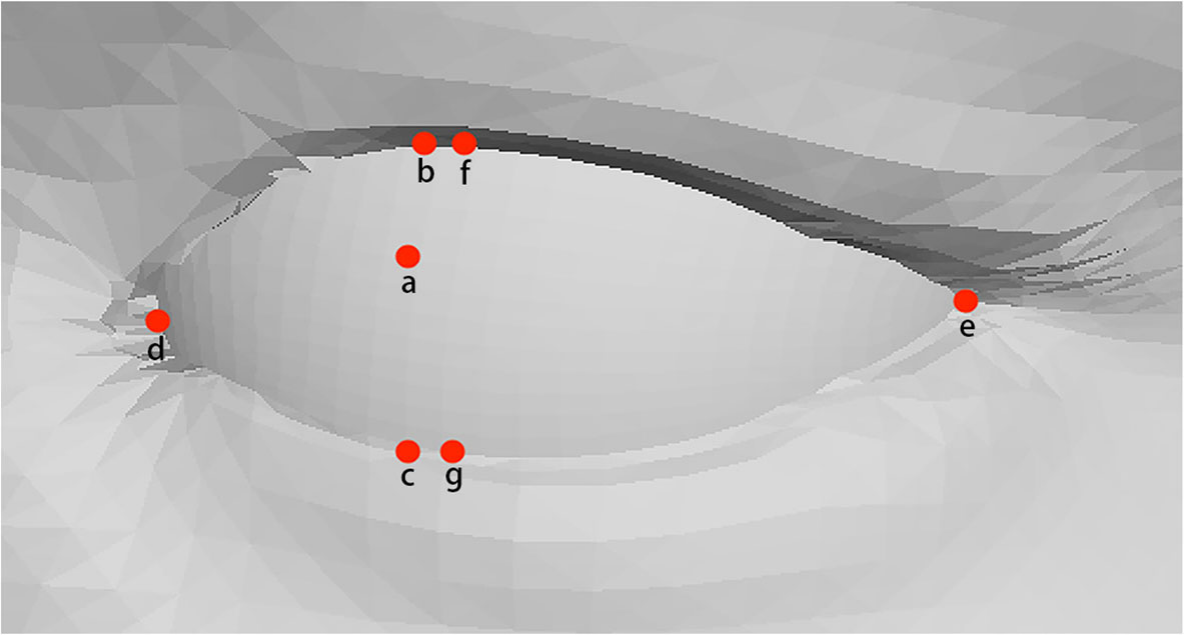
The key points corresponding to the key distance of the eye in the 3D model. **a**. The center of the eye. **b**. The upper eyelid margin used to count MRD1. **c**. The lower eyelid margin used to count MRD2. **d**. The inner canthus. **e**. The outer canthus. **f** and **g**. The upper and lower eyelid margin used to count the width of palpebral fissure

The 7 key distance measurements on the two-dimensional image are directly converted from the picture pixels and scale. In the three-dimensional measurement, we obtain the 3D coordinates of the 7 key points corresponding to the required key distance on the 3D model from the OBJ file, and use the fitting function to interpolate the surface in MATLAB to obtain the eyeball surface (Fig. 3). We use the ratio of the key point and scale ratio of the green discs to calculate the key distance.

Hitherto, we have completed the collection and statistics of 15 items of data for each eye, including 7 2D key distances, 7 3D key distances, and clinical surgery categories, i.e., the normalized ptosis data set size is 15 × 152.

After the dataset was established, we found that the construction of the clinical decision model faced a classification issue. The features of the classification were based on the patient’s multiple key distances in the eyes in two and three dimensions. The model prediction result could be categorized into clinical surgery.

Therefore, we applied various excellent classification models in the field of machine learning, such as SVM [16] and RF [17] after comparison of their classification accuracy, we selected the XGBoost model [18] based on the GBDT algorithm [19] for training and learning. Compared with other supervised learning algorithms, GBDT algorithm has fewer parameters, and the training process is faster and more stable. At the same time, compared with deep learning algorithm, the use of Feature Engineering and gradient promotion technology is more conducive to table data fitting. In recent years’ machine learning competition, the scheme based on GBDT method has got outstanding results.

### Gradient boosting decision tree (GBDT)

**Fig. 4:**
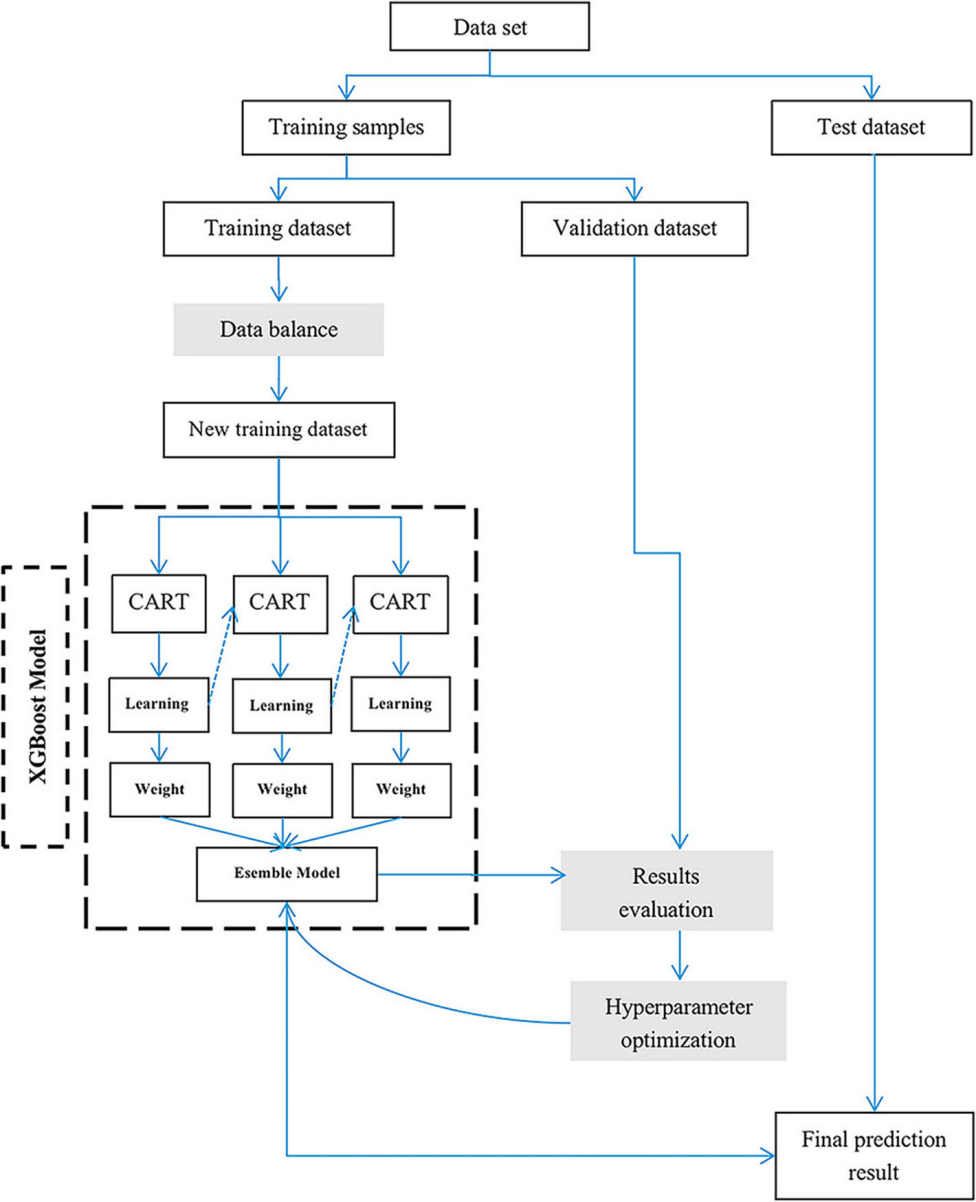
Schematic of the XGBoost model for training and learning. The balanced training set is used to learn the parameters in XGBoost model, and the validation set is used to optimize the hyperparameters of the model. Finally, the integrated model is used to test on the test set. In XGBoost model, CART is the base classifier, and the boosting strategy is adopted in the training process. By training a series of classifiers iteratively, the distribution of samples used by each classifier is related to the learning results of the previous round

The GBDT model is an integrated model (Fig. 4). The base classifier uses CART, and the integrated model is gradient boosting. The model can be expressed as follows: $F(x)={\sum}_{m=1}^M{\gamma}_m{h}_m(x)$. According to this idea, the prediction result of the m^th^ base classifier is: *F*_*m*_(*x*) = *F*_*m* − 1_(*x*) + *γ*_*m*_*h*_*m*_(*x*). The optimization goal of h (x) is to minimize the gap between the current prediction and y_i_: ${h}_m=\arg {\mathit{\min}}_h\sum \limits_{i=1}^nL\left({y}_i,\kern0.5em {F}_{m-1}\left({x}_i\right)+h\left({x}_i\right)\right)$.

Based on the GBDT algorithm, the XGBoost model mainly adds the following (Fig. 4):

1. Use the greedy algorithm to search for the optimal tree structure and select the split point with the largest gain to split each time.
2. A regular term is added to the cost function to control the complexity of the model.
3. After an iteration, the weight of the leaf nodes is multiplied by this coefficient, mainly to weaken the influence of each tree, such that there is more learning space behind.

### Experiment

The experiment is divided into three stages:

1. Formulate different feature selection schemes and select 3D or 2D features.
2. Perform the first binary classification of all patients’ eyes to assess whether the eye has ptosis disease
3. Perform the second binary classification of the eyes with ptosis. The classification results are categorized into two clinical plans: levator muscle resection and frontalis suspension.

### Experimental setting

1. The split ratio of training and test sets is 7:3.
2. Set three experimental schemes: use 2D distance alone, use 3D distance alone, and use two distances at the same time.
3. The XGBoost model refers to the XGBoost library in Python, and the parameters are adjusted as follows:

The current experiment is a binary classification problem, hence, according to this learning task, the learning goal during training is determined to be “binary: logistic”, which is the logistic regression of binary classification and output probability. After each enhancement step, the weight of the additional features can be obtained directly, and the feature weight can be reduced to make the enhancement process conservative. Adjustment of “ETA” (learning rate) renders the model lightweight and prevents over-fitting. The normalization algorithm is set to “tree” such that the weight of the new tree during training is the same as that of each fallen tree. Good values of “num_boost_round” and “nthread” ensure fast training of the model. We used the GridSearchCV module to perform the parameter adjustment process to perform grid search on various parameters and simulate the most appropriate parameter settings.

## Results

In this study, we used the five most common evaluation indicators in the binary classification problem: accuracy (ACC), precision (also called positive predictive value), recall (also known as sensitivity), F1-score, area under the curve (AUC) [20].

The five indicators are defined as follows:

1. Accuracy: Data correctly classified by the algorithm/amount of data entered into the algorithm;
2. We define:

TN: The algorithm predicts a negative example (N), which is the number of negative examples (N);

FP: The algorithm predicts a positive example (P), which is the number of negative examples (N);

FN: The algorithm predicts a negative example (N), which is the number of positive examples (P);

TP: The algorithm predicts a positive example (P), which is the number of positive examples (P).

Then we have: $precision=\frac{TP}{TP+ FP}$, $recall=\frac{TP}{TP+ FN}$, $F1=2\bullet precision\bullet \frac{recall}{precision+ recall}$.

To predict the occurrence of sickness, the recall rate should be under intensive focus, because if the real illness is not detected, the condition could worsen.

3. AUC value: AUC is defined as the area under the receiver operating characteristic (ROC) curve, which is based on two classification methods (cutoff value or decision threshold); the abscissa of the ROC curve is the false positive rate, and the ordinate is the true positive rate. AUC is a performance indicator that evaluates the advantages and disadvantages of the model.

The final results with 30 sets of data are summarized in the Table 1. For diagnostic discrimination, recall of “3D”, “2D” and “Both” were 0.875, 0.875 and 0.938 respectively. And precision of the three groups were 0.8333, 0.7778 and 1.0000 for the surgical procedure classification. Values of “Both” that combined 2D and 3D data were the highest in both classifications. We calculate the baseline indicators including age and sex, in which no significant difference was observed among different data sets during model training and verification.

**Table 1:** Diagnostic discrimination (whether or not suffering from ptosis) and of surgical procedure classification (using levator muscle resection or frontalis suspension)

Scheme	Diagnostic discrimination			Surgical procedure classification		
	3D	2D	Both	3D	2D	Both
ACC	0.8478	0.8043	0.8261	0.8182	0.8182	0.8182
AUC	0.833	0.759	0.795	0.817	0.773	0.833
F1-score	0.8889	0.8615	0.8824	0.8333	0.7000	0.8000
Precision	0.9032	0.8485	0.8333	0.8333	0.7778	1.0000
Recall	0.8750	0.8750	0.9375	0.8333	0.6364	0.6667

## Discussion

For diagnostic discrimination, we pay more attention to recall. For people with ptosis, it is better to have fewer wrong predictions. If the disease is not detected, the consequences could be dire. In the surgical procedure classification, we focus more on precision. The reason for this is that frontalis suspension is more costly, and it is often employed when levator muscle resection does not prove efficacious in clinical diagnosis and treatment. The higher precision is, the lower the predicted medical cost will be. These two situations are also consistent with the clinicians’ habits. The results demonstrate that, compared with the situation when 2D or 3D data are used alone, both recall and precision are greater when 2D and 3D data are used together. Thus, the prediction effect of our decision model is best when 2D data is combined with 3D data.

This experiment would further optimize the subjective diagnosis and treatment standards for ptosis in real clinical practice. The statistical analysis of multiple key distances in the patient’s eye would aid in establishing a targeted surgical decision model to achieve an end-to-end system for the surgical plan decision of ptosis.

For the diagnosis and treatment of ptosis, measurement of the MRD1, MRD2 from facial photographs by computer-assisted analysis has been previously described by Zachary, Michael and John [21]. However, the facial photographs used are 2D pictures, which may cause information loss. Also, only MRD1 and MRD2 are measured. To the best of our knowledge, several innovations proposed in our study are glamorous:

1. Optimize the existing “gold standard” [22, 23] by not judging the surgical plan based on a single distance or muscle strength measured manually, and reduce the error of the doctor’s subjective judgment. By designing an integrated dataset of 7 critical distances in the eye, we performed the most accurate regression classification using machine learning and high-accuracy surgical decisions. Concurrently, the image acquisition method is simple and effective for the existing troubles in the clinic that require doctors to measure by hand, making it a cost-effective repetition process. In children, collecting eye data is convenient and efficient.
2. Introduce the 3D key distance of the eye. Usually, in clinical diagnosis and treatment, doctors use a ruler to measure the eye data of patients as an approximate mathematical parameter. However, the key distances of the eye in our study are based on the curved surfaces of the eye frame, and using the 3D distance of the space improved the accuracy of the measurement. Given that it is impractical for doctors to obtain the 3D data directly in the actual diagnosis, we propose interdisciplinarity, which is integrating innovative computer technology and using the depth information of the face collected by the structured light camera to reconstruct the face. The obtained 3D model solves this problem. In the future, the 3D distance will be used in the diagnosis and treatment of other diseases, while mathematical models indicate medical significance.

In this study, we selected patient samples satisfied with the operation effect and used the chosen operation method by doctors in the real clinical practice as the “correct answer” training model. This led to controversial theories: 1. It cannot be proved that the correct operation method leads to the satisfactory operation effect—these patients may achieve excellent results irrespective of the surgery performed. 2. It is impossible to prove whether the patient’s satisfaction is due to the operation or the diagnosis and treatment experience—patients may believe that the eyelid appearance looks better after the procedure. Hence, this phenomenon cannot be used to explain that it is the appropriate surgery, as postulated in a previous study [24].

Although these controversies are intuitive, they are the precise focus of this article. Namely, a profound philosophical question is whether doctors should take rehabilitation or patients’ satisfaction as the purpose of the treatment. If we only think about the problem in the framework of “eyelid data meet a certain standard after surgery,” it could be easily confused by these controversies. As mentioned above, it is optimal to set the actual value of the outcome variable of the decision-making model as the patient evaluation. The series of judgment models were not based on the data to objective evaluation, and then from objective evaluation to subjective evaluation. This would make the decision invalid. On the contrary, we make a model directly from data for subjective evaluation. The ptosis addressed here is within the scope that not only paying attention to the real curative effect. Whether to use the term “happiness” is yet to be determined; however, considering the subjective feelings of patients in psychosomatic diseases is appropriate.

Despite the significant advantages mentioned above, there are some limitations: 1. Limited by the registration of the face recognition template, only seven sets of parameters were highly related to the key points of the human face in this study. Omissions may still exist, even if the measurement dimensions are more abundant than those in the current clinical application. Essentially, further optimization is needed after the relevant knowledge is surplus. 2. Because of no face data of patients in the retrospective research database, this study only collected prospective data. Thus, the lack of data resulted in the limited classification effect of the model. In the follow-up clinical practice, we will routinely collect facial 3D information to improve the accuracy of the model judgment.3. The ability of classification could be better if more accurate or more result-relevant data were provided for training. In our study, 3D data and seven eye parameters were introduced to provide data higher in accuracy. However, on account of the space and time, this paper does not involve the correlation study on parameters and results, which awaits future research.

## Conclusions

The 3D eye distances were initially introduced into the clinical diagnosis. Seven 2D and 3D ptosis-related eye data were integrated into a database to develop a model for ptosis surgery. Finally, a design for computer-assisted ptosis surgery was established. Our constructed model achieved best results when 2D and 3D data were employed jointly.

## Data Availability

The datasets during the current study available from the corresponding author on reasonable request.

## Abbreviations

2D: Two-dimensional
3D: Three-dimensional
ACC: Accuracy
AUC: Area under the curve
GBDT: Gradient boosting decision tree
ROC: Receiver operating characteristic
MRD: Margin reflex distance
